# Diversity alone does not reliably indicate the healthiness of an animal microbiome

**DOI:** 10.1093/ismejo/wrae133

**Published:** 2024-07-17

**Authors:** Claire E Williams, Tobin J Hammer, Candace L Williams

**Affiliations:** Department of Biology, University of Nevada, Reno, NV 89557, United States; Department of Ecology and Evolutionary Biology, University of California, Irvine, CA 92697, United States; Conservation Science, San Diego Zoo Wildlife Alliance, Escondido, CA 92027, United States

**Keywords:** Animal microbiome, host health, microbiome diversity

## Background

Animals harbor communities of microbes, known as microbiomes, in their gut and other body sites [1–3]. Given that these microbiomes are capable of mediating animal physiology and ecology, a key challenge is to determine the functional consequences of microbiome variation within host populations [4–8]. Such inter-individual microbiome variation has been observed across many animal species and tends to be particularly strong in wild populations [9–11]. Because most animal species are not experimentally tractable, a common approach is to use a microbiome’s diversity and composition to infer its “healthiness”—i.e. its effects on animal performance and fitness. Researchers often use two rules of thumb to evaluate whether a given microbiome is healthy or unhealthy for a given host: (1) higher alpha diversity indicates a healthy microbiome and (2) changes in microbiome beta diversity (interindividual variation) or composition indicate an unhealthy microbiome. These principles are extrapolated from findings about the human microbiome or the most closely related laboratory model, with little known about their applicability to wild animal populations.

We argue that drawing conclusions about an animal microbiome’s healthiness from diversity metrics alone is problematic. First, even for the deeply studied human microbiome, what aspects indicate a healthy state remains controversial [12, 13]. Even among healthy humans, microbiome diversity and composition vary across individuals and populations, shaped by factors like diet and environment [13]. Second, host species differ in baseline alpha diversity, absolute microbial abundance, longitudinal dynamics, and ecological function [14, 15]. For example, gut microbiomes of humans and closely related primates show divergent temporal dynamics, inter-individual variation, and the degree of co-diversification with hosts [13, 16]. Thus, rules developed in humans or lab models to infer host health outcomes from diversity metrics are more tenuous than is often assumed and may not translate to other species. Here, we weigh the evidence for and against the use of these rules in wild animal populations and propose an alternate path to identify healthy microbiomes. Although much of the literature we draw on concerns gut microbiomes, our arguments are equally applicable to microbiomes of skin or other body sites.

## Poorly tested principle 1: High alpha diversity indicates a healthy microbiome

Low alpha diversity is frequently depicted as indicating a disrupted microbiome with negative fitness consequences for the host (see Table S1). Loss of diversity is one of the standard hallmarks of “dysbiosis,” a microbiome state often assumed to precipitate disease [17–19] (but see literature [20, 21] for issues with this concept in general). For example, many studies have characterized the differences between the microbiomes of wild and captive animals, describing those of wild animals with higher alpha diversity as healthy, and those of captive animals with lower alpha diversity as disrupted. These characterizations imply that the loss of microbiota diversity underlies negative health and fitness outcomes associated with captivity (Table S1).

Principle 1 does have some theoretical and empirical support. Biodiversity–ecosystem function theory holds that high diversity increases ecosystem functioning via complementarity, sampling, and portfolio effects [22]. There is empirical evidence for this relationship in microbes; for example, synthetic community experiments have shown that high diversity increases productivity (though whether high microbiome productivity necessarily benefits host health is a separate and unresolved issue) [23]. In invasion ecology, the biotic resistance principle holds that diverse communities are less easily invaded by nonnative species [24]. Experiments on mice have provided support for this principle, finding that diverse gut microbiomes consume a broader array of nutrients, limiting resources available for pathogen growth [25]. Further, low diversity is sometimes correlated with disease in humans [19] and captive wildlife [26, 27].

Although high alpha diversity can be correlated with reduced disease, some conceptual frameworks suggest that it can instead be detrimental or neutral. Ecological models show that high diversity can destabilize microbiomes and/or require increased competitive interactions for stability, reducing overall community productivity [28]. Low diversity may actually be optimal in animals that normally harbor a low number of specialized and beneficial symbionts (e.g. bobtail squid, aphids). Such reduced partner diversity can be favored by selection, as evident when mutualisms evolve toward higher degrees of partner-specificity [29]. Further, some animals have largely transient microbiomes, for which differences in diversity may be irrelevant to host health [30].

There is empirical evidence that high alpha diversity does not always indicate a healthy microbiome. In humans, meta-analyses find that microbiome diversity is not consistently higher in healthy versus diseased patients [31, 32]. In fact, some diseases are characterized by high diversity, such as bacterial vaginosis [33], HIV/AIDS [34], and irritable bowel syndrome [35]. In other animals, a variety of diversity–disease relationships have been observed [36]. High diversity is associated with poor health states such as gastrointestinal distress in giant pandas [37] and chronic laminitis in horses [38]. Stressful hive conditions are linked to high microbiome diversity in honeybees [39], and antibiotics increase both microbiome diversity and mortality in weevils [40]. Even with regard to captivity, which is often depicted as reducing microbial diversity and consequently animal health, effects are highly idiosyncratic across animal taxa [41–43]. Altogether, there is no clear link between alpha diversity and health, either within or across species.

## Poorly tested principle 2: increased or altered beta diversity indicates an unhealthy microbiome

Beta diversity (i.e. interindividual variation or dispersion) is another key metric in most animal microbiome studies. Studies often describe altered microbiome composition or high interindividual variability as indicating an abnormal, unhealthy state (Table S1). The idea that microbiome stability is linked to host health has roots in the macrobial ecology literature. Healthy ecosystems are often defined as being resilient, returning to a particular state after stressful events [44]. For host-associated microbiomes, this idea may often hold true; the loss of beneficial microbial partners or gain of novel pathogens is likely to lower fitness. Indeed, shifts in microbiome composition and increased dispersion have been associated with host disease states in both humans and animal hosts [45].

Although shifts in microbiome composition or variability can be associated with disease, these shifts are not necessarily negative. One reason is that many microbes perform functionally redundant metabolic roles, such that the loss of one taxon may not cause the loss of a functional trait [46]. Another is that drastic changes in microbiome composition can be a part of normal developmental transitions such as maturation and aging [47, 48]. Further, microbiome deviations could increase resilience by allowing hosts to select for the most beneficial microbial partners in a given environment. Conversely, stressors that limit such variation may harm host health. For example, voles exposed to radiation in the Chernobyl exclusion zone had more temporally stable and constrained microbiome compositions than those not exposed to radiation [49]. The microbiome is generally acknowledged as a crucial source of host plasticity and rapid adaptation [50, 51], but for that to be true, the microbiome must be able to change.

Host species differ in the degree of interindividual and intraindividual (i.e. temporal) microbiome variability, and for most, we do not know what level of variability is normal [52, 53]. That level may differ among species. For example, *Acropora* corals have high microbiome turnover over time, whereas *Pocillopora* corals have stable microbiomes dominated by a single taxon [54]. High levels of gut microbiome variation have been seen in apparently healthy pandas [10, 53], meerkats [55], and bumble bees [56]. Therefore, interpreting whether beta diversity changes are pathological, neutral, or healthy for a given host is difficult.

## Methodological challenges

Our approaches to characterizing microbiomes are inherently limited in their ability to establish diversity–health relationships. Microbiomes of different body sites (e.g. gut versus skin) of the same host may have different relationships with health, and the methodology used to sample those sites may also introduce bias. For example, fecal samples are the most common way to sample the gut microbiome of wild animals, but fecal and gut microbiomes can exhibit substantially different alpha and beta diversity patterns across individuals [57, 58]. For 16S rRNA gene sequencing, the most common approach in current use, this is primarily due to its coarse resolution and lack of functional information [59]. Functionally diverse taxa may share the same or similar 16S rRNA gene sequences [60, 61]. Alternatively, distantly related bacteria with divergent 16S rRNA gene sequences may fulfill similar metabolic roles in a microbiome [46]. Technical artifacts are also rampant, including reagent contamination and biases in DNA extraction and sequencing, which can affect the comparability of different studies. In addition, the choice of metric used to characterize the microbiome could confound or bias associations with host health. For example, two microbiomes with similar species richness may have very different effects on hosts if their compositions differ. Moreover, effects of common versus rare microbes will be weighted differently depending on whether the beta diversity metric in use incorporates relative abundances. There is no single, standardized set of metrics, and there is no ecologically grounded basis for which ones should be most closely tied to host health [59].

## Future directions

Identifying general rules about what makes a healthy microbiome is a key goal for the field. However, in most animal species, we currently do not know what compositions, levels of diversity, or degrees of change are within the sphere of a healthy microbiome. To accomplish this, we need to understand multiple key aspects of a species’ microbiome: its members, temporal dynamics, and its combined resulting function for the host.

**Figure 1:**
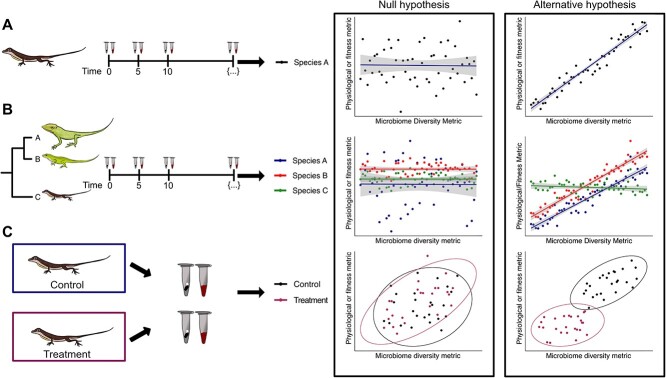
(A) Longitudinal sampling of both host physiological/fitness-related metrics and microbiome composition should be used to elucidate the relationship (or lack thereof) between the two in a given taxon. (B) This relationship can then be tested in related species, ultimately expanding to more distantly related taxa, for which the relationship may or may not hold. (C) Experimental manipulation of the gut microbiota (e.g. through antibiotic administration, germ-free experiments, or fecal transplants) could be used to test the causative nature of the relationship.

Among closely related species, diversity-health rules might indeed exist and be generalizable. However, these relationships must be established and tested. For example, a series of studies could start by longitudinally monitoring both the microbiome and some aspect of host fitness or a relevant physiological indicator in a given species (Fig. 1). Data describing what both healthy and unhealthy animals’ microbiomes look like across time could enable a species-specific model of microbiome healthiness in terms of composition and variability. Using multi-omic data (e.g. shotgun metagenomics, transcriptomics, and metabolomics) can also help us understand, which levels of microbiome diversity, be they taxonomic or functional, may best predict host physiology and fitness. From there, studies could focus on testing whether this model holds in closely related species. Systematically comparing these patterns to those in more distantly related taxa could then determine the extent of their generalizability. Ultimately, manipulative experiments—although not possible for many species—are necessary to determine the causality of these relationships.

We argue that it is time to move away from extrapolating existing (and often equivocal) information from humans and lab models, and toward embracing the diversity of ways in which wild animals interact with their microbial partners. We know that the alpha and beta microbiome diversity metrics in current use do not consistently predict host health status. This is not particularly surprising, given that these metrics imperfectly capture what matters most to a host: microbiome function. Further, variation in microbiome dynamics across the tree of life means that truly general diversity–health relationships may not exist. For many wild populations, we may never achieve a causal understanding of how microbiome variation affects host health. Regardless, host health and physiology metrics can and should be directly measured alongside the microbiome. Determining which microbiome features predict host health within and (perhaps) across species will help us better understand and manage animal–microbe relationships.

## Supplementary Material

TableS1_ISME_wrae133

## Data Availability

Data sharing is not applicable to this manuscript as no datasets were generated.
